# Numerical study of the effect of liquid compressibility on acoustic droplet vaporization

**DOI:** 10.1016/j.ultsonch.2021.105769

**Published:** 2021-09-25

**Authors:** Sukwon Park, Gihun Son

**Affiliations:** Department of Mechanical Engineering, Sogang University, Seoul, South Korea

**Keywords:** Acoustic droplet vaporization, Bubble growth, Bubble rebound, Liquid compressibility, Shock wave, Threshold

## Abstract

In acoustic droplet vaporization (ADV), a cavitated bubble grows and collapses depending on the pressure amplitude of the acoustic pulse. During the bubble collapse, the surrounding liquid is compressed to high pressure, and liquid compressibility can have a significant impact on bubble behavior and ADV threshold. In this work, a one-dimensional numerical model considering liquid compressibility is presented for ADV of a volatile microdroplet, extending our previous Rayleigh-Plesset based model [Ultrason. Chem. 71 (2021) 105361]. The numerical results for bubble motion and liquid energy change in ADV show that the liquid compressibility highly inhibits bubble growth during bubble collapse and rebound, especially under high acoustic frequency conditions. The liquid compressibility effect on the ADV threshold is quantified with varying acoustic frequencies and amplitudes.

## Nomenclature

c: specific heat
C: sound speed
$f_{a}$: frequency of an acoustic pulse
G: phase-change mass flux at the bubble surface
$i_{\mathrm{bd}}$: latent heat of vaporization
n: constant in the Tait equation
p: pressure
$P_{a}$: pressure amplitude of an acoustic pulse
r: radial coordinate
R: radius
$R_{g}$: gas constant
t: time
T: temperature
u: radial velocity
v: velocity in moving coordinate, $u-{\mathrm{dR}}_{b}/\mathrm{dt}$

**Greek symbols**
α: step function
γ: specific heat ratio
Γ: constant in the Tait equation
λ: thermal conductivity
μ: dynamic viscosity
ρ: density
σ: surface tension coefficient
ξ: moving coordinate, $r-R_{b}$

**Subscripts**
b,d: bubble, droplet
c: critical
l: liquid
L: domain boundary, r=L
o: initial
sat: saturation
w: water
∞: ambient
0: domain center, r=0

## 1 Introduction

Acoustic droplet vaporization (ADV) is emerging in the field of non-invasive targeted therapy and diagnosis, as reviewed in Refs. [1], [2]. Nanodroplets are vaporized into microbubbles when the amplitude of ultrasonic pulses exceeds a certain intensity called ADV threshold. An ultrasound-triggered bubble, which oscillates with alternating the positive and negative pressure portion of the pulses, can rapidly collapse with or without rebounds depending on the acoustic amplitude [3], [4], [5], [6], [7]. During the rapid bubble collapse, the liquid region near the bubble is also compressed to a high pressure above the critical pressure, resulting in a shock wave emission [8]. The liquid compressibility can affect the bubble behavior after collapse and thus ADV threshold, as indicated in Refs. [6], [7], [9]. However, a general numerical model including the liquid compressibility effect on ADV is lacking in the literature.

Numerical studies of ADV were performed using Rayleigh-Plesset (RP) equations [6], [7], [10], [11], [12] and multidimensional numerical models [13], [14], [15]. Although the multidimensional models can be applied to non-spherical bubble growth in ADV and the interactions of ADV bubbles and walls, they are not efficient for predicting the ADV threshold because very fine computational meshes are required to treat the initial bubble nuclei. Most of numerical models for ADV were developed from one-dimensional conservation equations for spherical bubble growth. The RP equation was derived by integrating the radial momentum equations for droplet and surrounding water regions with the interface conditions at the bubble and droplet surfaces. Assuming that the bubble was an ideal gas, the bubble growth and survival due to acoustic pulses were investigated. Recently, Park and Son [7] improved the RP equation for ADV using the van de Waals (VDW) equation of state applicable to the supercritical state of a collapsing and rebounding bubble. However, all studies of ADV mentioned above did not consider liquid compressibility.

The effect of liquid compressibility was included in several studies investigating the bubble growth and collapse in infinite water. Gilmore [16] and Keller-Miksis [17] equations, which are a kind of RP equation applied to a weakly compressible liquid, were widely used to investigate bubble motions in acoustic fields. These equations can be derived from the integration of the radial momentum equation between the bubble surface and the domain boundary introducing a velocity potential, as described in Ref. [17]. The velocity potential cannot be determined directly from the continuity equation as in the incompressible liquid case, but is expressed as a general solution of the wave equation approximated from the continuity and momentum equations. In the compressible RP equations, which are derived by eliminating the time derivative of the velocity potential, the liquid compressibility effect is considered in terms of the sound speed and the time derivative of the boundary pressure, which are not shown in the incompressible RP equation. Although the compressible RP equations were applied to bubble growth and collapse in infinite water, they were not applied to the ADV case, in which the compressible RP equations cannot be easily derived for the droplet and water regions without including unknown variables such as the time derivatives of the droplet surface pressures or the velocity potentials in the two regions. As an alternative to the compressible RP-based model, numerical models directly solving the conservation equation without using the weak liquid compressibility assumption were proposed in several studies [18], [19], [20], [21] for bubble motion in a compressible liquid. They investigated the bubble behaviors triggered by acoustic pressure waves and the pressure wave transition during bubble collapse. However, the methods were not extended to ADV which includes the droplet and water regions.

In this work, a numerical method for bubble growth in ADV considering liquid compressibility is developed by extending our previous RP based model [7]. One-dimensional spherical conservation equations of mass, momentum and energy are directly solved for bubble motion in ADV. The present model is applied to the ADV experimental cases of Aliabouzar et al. [22].

## 2 Numerical modeling

**Fig. 1:**
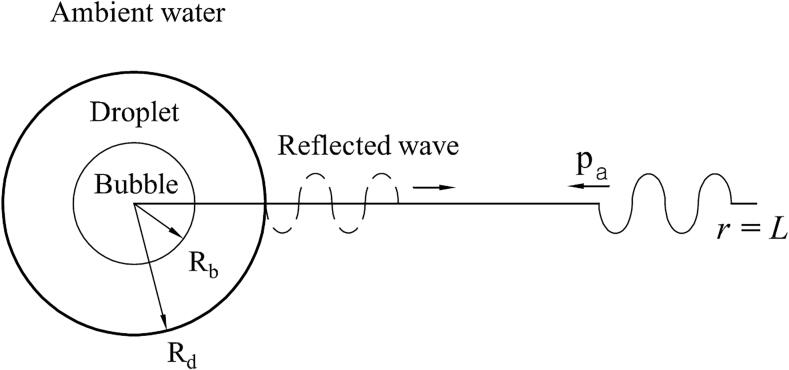
Configuration of acoustic vaporization in compressible liquid (droplet and ambient water) regions.

Fig. 1 depicts the configuration of acoustic vaporization in compressible liquid (droplet and ambient water) regions. A spherical dodecafluoropentane (DDFP) microdroplet with a radius of $R_{d}$ and a boiling point of 29 °C at 1 atm is placed in the ambient water. An acoustic pressure wave of $p_{a}$ is generated at the domain boundary of r=L, and transmitted through the droplet at r=0. A DDFP bubble with a radius of $R_{b}$ is assumed to grow at the droplet center.

### 2.1 Governing equations

Assuming that the flows are spherically symmetric, the conservation equations for compressible liquid regions are expressed as

(1) $$\frac{\partial r^{2}\rho_{l}}{\partial t}+\frac{\partial r^{2}u_{l}\rho_{l}}{\partial r}=0$$

(2) $$\rho_{l}(\frac{\partial u_{l}}{\partial t}+u_{l}\frac{\partial u_{l}}{\partial r})=-\frac{\partial p_{l}}{\partial r}+\frac{4}{3}\frac{1}{r^{2}}\frac{\partial }{\partial r}r^{2}\mu_{l}(\frac{\partial u_{l}}{\partial r}-\frac{u_{l}}{r})+\frac{4}{3}\frac{\mu_{l}}{r}(\frac{\partial u_{l}}{\partial r}-\frac{u_{l}}{r})$$

(3) $${\left(\rho c\right)}_{l}(\frac{\partial T_{l}}{\partial t}+u_{l}\frac{\partial T_{l}}{\partial r})=-\frac{p_{l}}{r^{2}}\frac{\partial r^{2}u_{l}}{\partial r}+\frac{1}{r^{2}}\frac{\partial }{\partial r}r^{2}\lambda_{l}\frac{\partial T_{l}}{\partial r}+\frac{4}{3}\mu_{l}{\left(\frac{\partial u_{l}}{\partial r}-\frac{u_{l}}{r}\right)}^{2}$$

where the subscript l denotes the droplet region (d) if $R_{b}<r<R_{d}$ and the ambient water region (w) if $R_{d}<r<L$. In Eq. (1), consisting of the unsteady and mass flux terms, the liquid velocity cannot be easily evaluated as a function of the bubble radius $R_{b}$ due to the liquid compressibility condition ($D\rho_{l}/\mathrm{Dt}\;\neq \;0$). Eq. (2) contains not only the inertia and pressure terms, but also two terms of viscous stress, which do not appear in the incompressible fluid case. Each term in Eq. (3) represents internal energy change, pressure work, heat transfer and viscous dissipation, respectively.

The equations for each liquid region are combined by the following conditions at $r=R_{d}$:

(4) $$u_{d}=u_{w}$$

(5) $$p_{d}-p_{w}=\frac{2\sigma_{\mathrm{dw}}}{r}+\mu_{d}\frac{4}{3}\left(\frac{\partial u_{d}}{\partial r}-\frac{u_{d}}{r}\right)\;-\mu_{w}\frac{4}{3}\left(\frac{\partial u_{w}}{\partial r}-\frac{u_{w}}{r}\right)$$

(6) $$T_{d}=T_{w}$$

(7) $$\lambda_{d}\frac{\partial T_{d}}{\partial r}=\lambda_{w}\frac{\partial T_{w}}{\partial r}$$

Assuming uniform pressure and temperature distributions inside the bubble [6], [10], [11], [12], the boundary conditions at $r=R_{b}$ are expressed as

(8) $$\frac{{\mathrm{dR}}_{b}}{\mathrm{dt}}=u_{d}+\frac{G}{\rho_{d}}$$

(9) $$p_{d}=p_{b}-\frac{2\sigma_{\mathrm{bd}}}{r}+(\frac{1}{\rho_{b}}-\frac{1}{\rho_{d}})G^{2}+\frac{4}{3}\mu_{d}(\frac{\partial u_{d}}{\partial r}-\frac{u_{d}}{r})$$

(10) $$T_{d}=T_{b}$$

In Eq. (8), $u_{d}$ is the droplet-side velocity at the bubble surface, which can be obtained from Eq. (2), and is not equal to the bubble surface velocity ${\mathrm{dR}}_{b}/\mathrm{dt}$ due to the phase-change rate $G/\rho_{d}$. The phase-change mass flux *G* at $r=R_{b}$ can be written as

(11) $$G=\frac{1}{i_{\mathrm{bd}}}\lambda_{d}\frac{\partial T_{d}}{\partial r}$$

The boundary conditions at r=L are specified as

(12) $$p_{w}=p_{\infty }+p_{a,L}$$

(13) $$T_{w}=T_{\infty }$$

where $p_{a,L}$ is the acoustic pressure imposed at the domain boundary.

Assuming that the bubble density is uniform inside the bubble, $\rho_{b}$ is determined by the bubble mass conservation,

(14) $$\frac{d}{\mathrm{dt}}\left(\rho_{b}R_{b}^{3}\right)=3R_{b}^{2}G\;\;\;\mathrm{or}\;\;\;\frac{d\rho_{b}}{\mathrm{dt}}=\frac{3}{R_{b}}\left(G-\rho_{b}\frac{{\mathrm{dR}}_{b}}{\mathrm{dt}}\right)$$

The VDW Eq. (15) is used to consider the bubble compressibility and supercritical state of a collapsing and rebounding bubble. It is combined with Clausius–Clapeyron Eq. (16) and $\rho_{b}$ from Eq. (14) to determine the bubble temperature $T_{b}$ and pressure $p_{b}$, as described in our previous study [7].

(15) $$p_{b}=\frac{\rho_{b}R_{g}T_{b}}{1-b\rho_{b}}-a\rho_{b}^{2}$$

(16) $$p_{b}=p_{\infty }\exp[\frac{i_{\mathrm{bd}}}{R_{g}}(\frac{1}{T_{b,\infty }}-\frac{1}{T_{b}})]$$

where the constants *a* and *b* are obtained from the critical properties [23]. The bubble pressure $p_{b}$ is used as the boundary condition to determine the droplet-side pressure at the bubble surface, as given by Eq. (9). When the bubble pressure exceeds the critical value $p_{c}$, we assume that *G* becomes zero and the bubble is isentropic during the supercritical state as done by Refs. [7], [24].

**Fig. 2:**
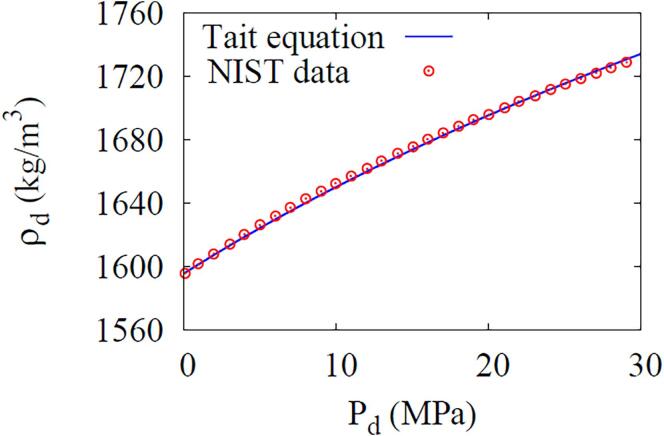
Relationship between pressure and density of DDFP liquid using the Tait equation.

The liquid density $\rho_{l}$ is obtained from Eq. (1) and the liquid pressure $p_{l}$ is determined from the Tait equation,

(17) $$p_{l}=\Gamma_{l}[{\left(\frac{\rho_{l}}{\rho_{l,\infty }}\right)}^{n_{l}}-1]+p_{\infty }$$

where the constants are evaluated as $\Gamma_{w}=331\;\text{MPa}$ and $n_{w}=7.15$ for water [25]. For the DDFP liquid, $\Gamma_{d}=36.4\;\text{MPa}$ and $n_{d}=7.23$ are determined by fitting the NIST data [26] at 303K as depicted in Fig. 2 and referring to the sound speed of $C_{d}=406\;\text{m}/\text{s}$ reported in Ref. [27].

Introducing the moving coordinate $\xi =r-R_{b}$ and using the level-set method [5], [14], [21] so that the conservation equations implicitly satisfies the interface conditions at $r=R_{d}$, given by Eqs. (4), (5), (6), (7), the conservation Eqs. (1), (2), (3) can be rewritten for the liquid regions as

(18) $$\frac{\partial r^{2}\rho_{l}}{\partial t}+\frac{\partial r^{2}v_{l}\rho_{l}}{\partial \xi }=0$$

(19) $$\rho_{l}(\frac{\partial u_{l}}{\partial t}+v_{l}\frac{\partial u_{l}}{\partial \xi })=-[\frac{\partial p_{l}}{\partial \xi }+\frac{2\sigma_{\mathrm{dw}}}{R_{d}}\frac{d\alpha (r-R_{d})}{\mathrm{dr}}]+\frac{1}{r^{2}}\frac{4}{3}\frac{\partial }{\partial \xi }r^{2}\mu_{l}(\frac{\partial u_{l}}{\partial \xi }-\frac{u_{l}}{r})+\frac{1}{r^{2}}\frac{4}{3}r\mu_{l}(\frac{\partial u_{l}}{\partial \xi }-\frac{u_{l}}{r})$$

(20) $${\left(\rho c\right)}_{l}(\frac{\partial T_{l}}{\partial t}+v_{l}\frac{\partial T_{l}}{\partial \xi })=-\frac{p_{l}}{r^{2}}\frac{\partial r^{2}u_{l}}{\partial \xi }+\frac{1}{r^{2}}\frac{\partial }{\partial \xi }r^{2}\lambda_{l}\frac{\partial T_{l}}{\partial \xi }+\frac{1}{r^{2}}\frac{4}{3}r^{2}\mu_{l}{\left(\frac{\partial u_{l}}{\partial \xi }-\frac{u_{l}}{r}\right)}^{2}$$

where the relative velocity $v_{l}$ and the step function α are defined as

(21) $$v_{l}=u_{l}-\frac{{\mathrm{dR}}_{b}}{\mathrm{dt}}$$

(22) α(r)=0ifr⩽0

(23) α(r)=1ifr>0

For a two-phase mixture cell that includes the droplet surface ($r=R_{d}$), the properties $\rho_{l},{\left(\rho c\right)}_{l},\mu_{l}$ and $\lambda_{l}$ are evaluated by interpolation of the droplet and water properties using the level-set method [5], [14], [21]. We discretize the conservation equations in a staggered grid system using the second-order ENO and central difference schemes for spatial discretization of the convection and diffusion terms, and a third-order TVD Runge–Kutta method [28] with an adaptive time step for temporal discretization of the conservation equations and other unsteady equations.

Eqs. (18), (19), (20) are solved for $\rho_{l},u_{l}$ and $T_{l}$ with the boundary conditions, given by Eqs. (9), (10), (11), (12), (13), which includes $R_{b},\rho_{b},p_{b}$ and $T_{b}$ to be determined from Eqs. (8), (14), (15), (16), respectively, and $R_{d}$ from $u_{l}$ interpolated at $r=R_{d}$.

### 2.2 Computational conditions

Computations of ADV are performed for a DDFP microdroplet immersed in the ambient water at $p_{\infty }$ = 1 atm and $T_{\infty }=310\;\text{K}$. We choose the initial droplet radius of $R_{\mathrm{do}}=0.47\;\mu \text{m}$ from the ADV experimental condition of Aliabouzar et al. [22] and a large computational domain of L=2.74mm to prevent reflection of acoustic waves at the boundary (r=L). The properties of DDFP and water are obtained from our previous work [7]. The acoustic pressure pulse imposed at the domain boundary (r=L) is expressed as

(24) $$p_{a,L}=P_{a,L}\sin(2\pi f_{a}t)\;\mathrm{if}\;t⩽N_{a}/f_{a}$$

(25) $$p_{a,L}=0\;\;\;\;\;\;\;\mathrm{if}\;t>N_{a}/f_{a}$$

Here, $P_{a,L}$ and frequency $f_{a}$ vary in this work keeping $N_{a}=8$ based on Ref. [22].

For a computational domain of $R_{b}<r<L$ (or 0 <ξ<$L-R_{b}$), uniform fine meshes of size $\Delta \xi_{o}$ are chosen near the droplet ($0<\xi <20R_{\mathrm{do}}$), nonuniform meshes with sizes increasing by a factor of 1.0004 in the middle region ($20R_{\mathrm{do}}<\xi <1600R_{\mathrm{do}}$), and uniform meshes in the outer region ($1600R_{\mathrm{do}}<\xi <L-R_{b}$).

**Fig. 3:**
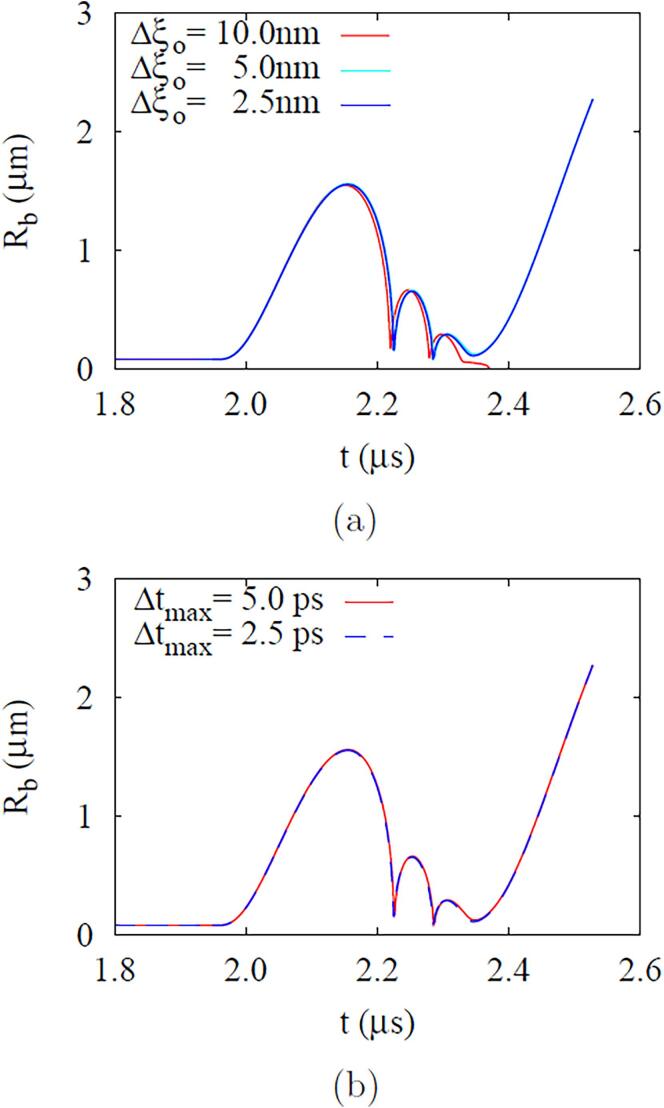
Resolution test for ADV calculation at $R_{\mathrm{do}}=0.47\;\mu \text{m},f_{a}=2.25\;\text{MHz}$ and $P_{a,L}=10.4\;\text{kPa}$: (a) mesh size and (b) time step.

Convergence tests for mesh size and time step are conducted to determine the mesh size $\Delta \xi_{o}$ and time step Δt, as presented in Fig. 3. The numerical results for bubble growth with different mesh sizes are converged for $\Delta \xi_{o}⩽5\;\text{nm}$. The time step convergence test with adaptive time steps with $\Delta t_{\max}=5\;\text{ps}$ and $\Delta t_{\max}=2.5\;\text{ps}$, shows no difference in the results with two time steps. Therefore, $\Delta \xi_{o}=5\;\text{nm}$ and the adaptive time step with $\Delta t_{\max}=5\;\text{ps}$ are used in this work.

## 3 Results and discussion

### 3.1 Acoustic pressure amplification in compressible liquids

**Fig. 4:**
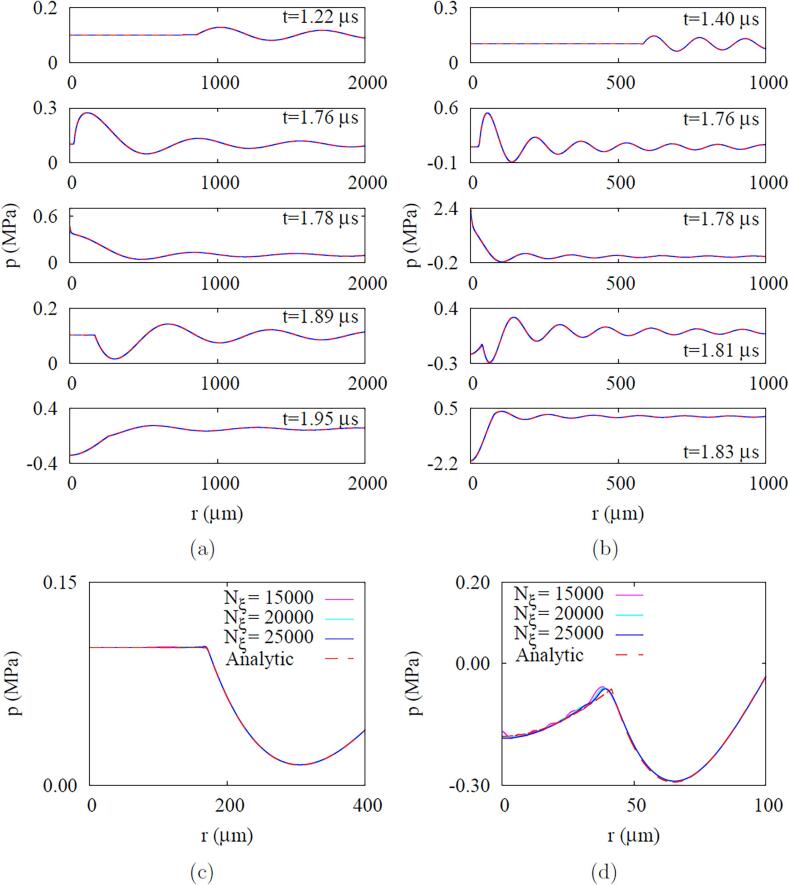
Effect of frequency $f_{a}$ on the acoustic wave propagation in water without a droplet at $P_{a,L}=10\;\text{kPa}$: (a) $f_{a}=2.25\;\text{MHz}$ , (b) $f_{a}=10\;\text{MHz}$, (c) and (d) influence of grid number on the numerical result of the fourth figure in (a) and (b), respectively. In figures a and b, the blue solid lines and the red dash lines indicate the numerical and analytic predictions, respectively.

Computations are first performed on acoustic pressure waves traveling in the ambient water without a droplet to validate the present model for compressible flows. The domain size chosen with L=2.74mm is 4 times the wavelength ($C_{w,\infty }/f_{a}$) for $f_{a}=2.25\;\text{MHz}$ and almost 18 times the wavelength for $f_{a}=10\;\text{MHz}$. Fig. 4 shows the evolution of pressure waves generated under the acoustic conditions of $P_{a,L}=10\;\text{kPa}$ and $f_{a}=2.25\;\text{MHz}$ or 10MHz at r=L. The pressure waves travel through the ambient water with $C_{w,\infty }=1540\;\text{m}/\text{s}$ and reach the domain center (r=0) at t=1.775μs. Assuming that the water viscosity is negligible and the pressure amplitude is small, the analytic solution of the linearized pressure wave equation for a one-dimensional spherical case can be derived as [21]

(26) $$p\left(t,r\right)=p_{\infty }+P_{a,L}\frac{L}{r}\overset{\infty }{\underset{n=1}{\sum }}\left[E\left(t+\frac{r-\left(2n-1\right)L}{C_{w,\infty }}\right)\;\;\;+E\left(t+\frac{r+\left(2n-1\right)L}{C_{w,\infty }}\right)\right]$$

where

(27) $$E(t)=\alpha (t)\sin(2\pi f_{a}t)$$

The pressure at r=0 is evaluated as

(28) $$p\left(t,0\right)=p_{\infty }+P_{a,0}\overset{\infty }{\underset{n=1}{\sum }}\left(\alpha \left[t-\frac{\left(2n-1\right)L}{C_{w,\infty }}\right]\;\;\;\times \cos\left[2\pi f(t-\frac{\left(2n-1\right)L}{C_{w,\infty }})\right]\right)$$

where

(29) $$P_{a,0}=P_{a,L}\frac{4\pi f_{a}L}{C_{w,\infty }}$$

The present numerical results have no difference from the analytical predictions given by Eq. (26). As the acoustic wave approaches r=0, its magnitude is amplified to $50P_{a,L}$ for $f_{a}=2.25\;\text{MHz}$ and $220P_{a,L}$ for $f_{a}=10\;\text{MHz}$. Since the acoustic amplitude varies with the radial location in a compressible liquid, we choose the reference amplitude from the amplitude $P_{a,0}$ at r=0 in the absence of a droplet. In a typical experimental setup for ADV, the acoustic amplitude was measured using a hydrophone at the underwater focal spot of an ultrasonic transducer, and then a droplet was placed at the focal spot [29], [30]. This selection of acoustic amplitude as $P_{a,0}$ seems to be consistent with the hydrophone measurement method. It is noted that a large number of mesh points of $N_{\xi }=25,000$ are used in the present computations to avoid numerical oscillations occurring at high acoustic frequencies, as depicted in Fig. 4d for $f_{a}=10\;\text{MHz}$.

**Fig. 5:**
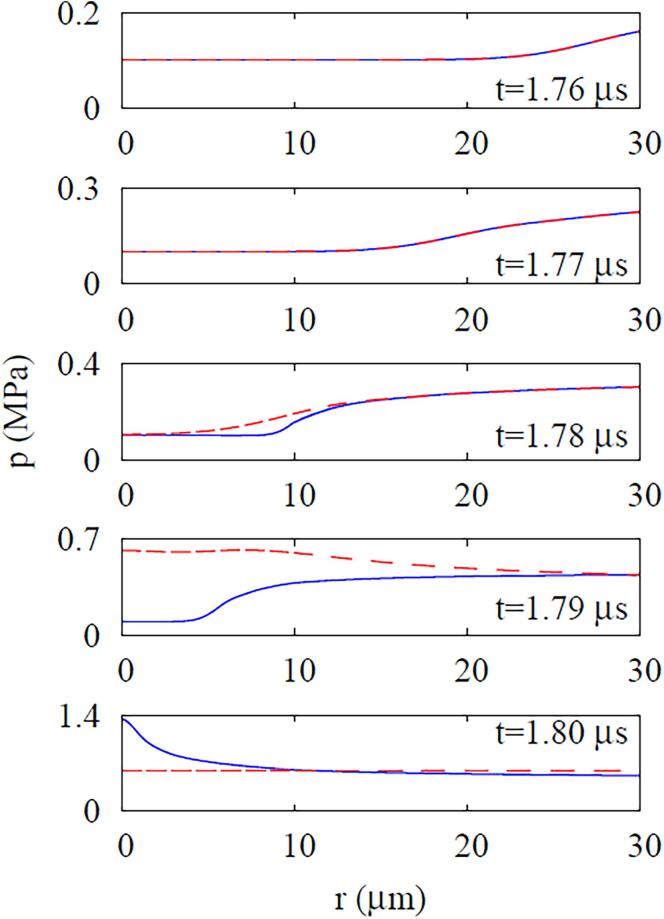
Acoustic wave propagation at $f_{a}=2.25\;\text{MHz}$ and $P_{a,0}=0.5\;\text{MPa}$. In the figure, the blue solid lines and the red dash lines indicate the cases with and without a droplet ($R_{\mathrm{do}}=10\;\mu \text{m}$), respectively.

**Fig. 6:**
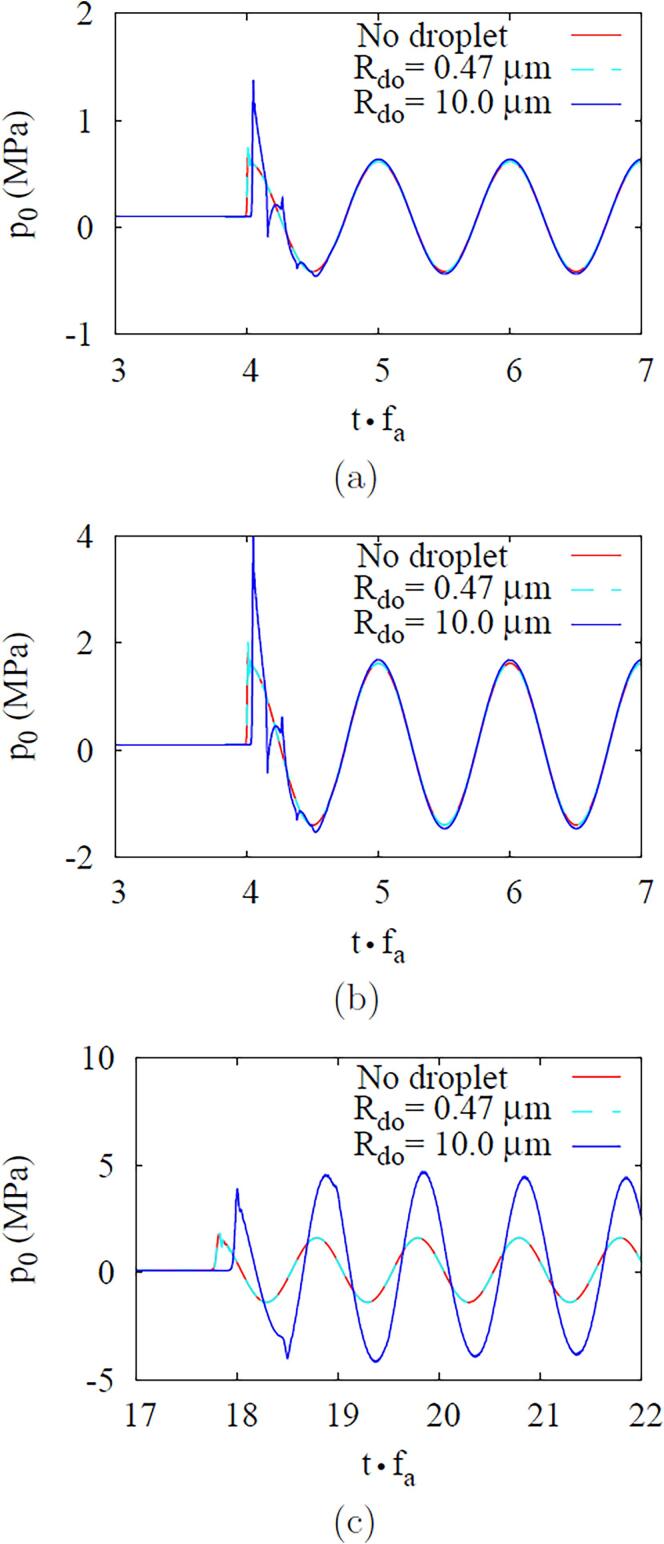
Effects of droplet size $R_{\mathrm{do}}$ and frequency $f_{a}$ on the acoustic wave amplification: (a) $f_{a}=2.25\;\text{MHz}$ and $P_{a,0}=0.5\;\text{MPa}$, (b) $f_{a}=2.25\;\text{MHz}$ and $P_{a,0}=1.5\;\text{MPa}$, and (c) $f_{a}=10\;\text{MHz}$ and $P_{a,0}=0.5\;\text{MPa}$.

Calculations are next made for an acoustic pressure wave traveling through a DDFP droplet immersed in the ambient water without bubble nucleation. The results are plotted in Fig. 5, Fig. 6. Although the presence of a droplet affects the pressure wave passing the droplet region during the first cycle, as seen in Fig. 5, its influence on the pressure $p_{0}$ at r=0, except for the first cycle, is insignificant at $f_{a}=2.25\;\text{MHz}$ regardless of the droplet radius and acoustic amplitude, as depicted in Fig. 6, Fig. 6b. The droplet effect on the pressure amplification is pronounced for $f_{a}=10\;\text{MHz}$ and $R_{\mathrm{do}}=10\;\mu \text{m}$, as seen in Fig. 6c. This is expected from Eq. (29), which indicates the pressure amplification is proportional to $f_{a}$ and inversely proportional to the sound speed. Since the sound speed of DDFP droplet is lower than that of ambient water, the pressure amplification in the droplet increases as the droplet becomes larger, especially for the high acoustic frequency case of $f_{a}=10\;\text{MHz}$. This is consistent with the observation in Ref. [27] that the amplification of pressure wave is proportional to $f_{a}$ with increasing the droplet size.

### 3.2 Acoustic vaporization in compressible liquids

We now consider acoustic vaporization of a DDFP droplet with $R_{\mathrm{do}}=0.47\;\mu \text{m}$ in the ambient water at $T_{\infty }=310\;\text{K}$ and $p_{\infty }=1\;\text{atm}$. An acoustic pressure wave generated at the domain boundary (r=L) with $P_{a,L}=10\;\text{kPa}$ and $f_{a}=2.25\;\text{MHz}$ is amplified to $P_{a,0}=0.5\;\text{MPa}$ when it reaches the domain center (r=0), as described in Section 3.1. A bubble with an initial radius of $R_{\mathrm{bo}}$ is assumed to form at the droplet center. We choose $R_{\mathrm{bo}}=80\;\text{nm}$, which was also used in Refs. [7], [12]. Since the effect of $R_{\mathrm{bo}}$ on ADV was investigated in our previous study [7], we focus on the effect of liquid compressibility on ADV in this work.

Considering the Laplace pressure and acoustic pulsing condition, the initial bubble pressure is evaluated as

(30) $$p_{\mathrm{bo}}=p_{\infty }+\frac{2\sigma_{\mathrm{bd}}}{R_{\mathrm{bo}}}+\frac{2\sigma_{\mathrm{dw}}}{R_{\mathrm{do}}}+p_{a,0}$$

where $p_{a,0}$ is the acoustic pressure at the droplet center, as depicted in Fig. 6. Assuming that bubble nucleation occurs in thermal equilibrium with the ambient condition, $p_{\mathrm{bo}}=p_{\mathrm{sat}}(T_{\infty })$, and using the properties of $p_{\mathrm{bo}}=0.131\mathrm{MPa},\sigma_{\mathrm{bd}}=9.5\times 10^{-3}\;\text{N}/\text{m}$ and $\sigma_{\mathrm{dw}}=5\times 10^{-2}\;\text{N}/\text{m}$ [7], we have $p_{a,0}=-0.421\;\text{MPa}$ from Eq. (30).

**Fig. 7:**
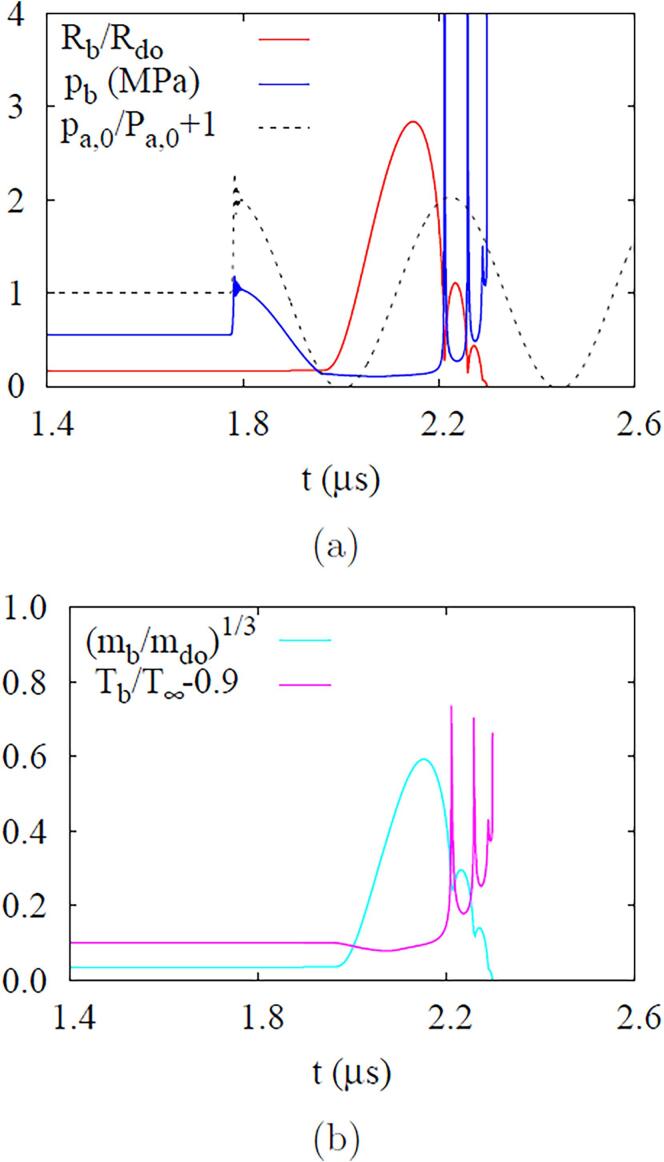
ADV at $f_{a}=2.25\;\text{MHz}$ and $P_{a,0}=0.5\;\text{MPa}$: temporal variations of (a) bubble radius, pressure, (b) temperature and mass.

Fig. 7 shows bubble growth and collapse in ADV at $p_{a,0}=0.5\;\text{MPa}$ and $f_{a}=2.25\;\text{MHa}$. Bubble nucleation is assumed to occur at t=1.96μs, when $p_{a,0}=-0.421\;\text{MPa}$. During the negative pressure pulsing period after the bubble nucleation, $p_{b}$ continuously decreases and the bubble radius increases to $R_{b}=1.35\;\mu \text{m}$. As the bubble temperature $T_{b}$ is reduced below $T_{\infty }$, the evaporation at $r=R_{b}$ increases the bubble mass $m_{b}$. Thereafter, during the positive pulsing period, $R_{b}$ decreases to 0.13μm. While $T_{b}$ increases above $T_{\infty }$, the condensation at $r=R_{b}$ reduces $m_{b}$. As the bubble further compresses or collapses due to the positive pulsing, $p_{b}$ exceeds the critical pressure $p_{c}=2.25\;\text{MPa}$ and then rapidly rises to a very high pressure at the supercritical state without phase change. When the liquid pressure increases near the collapsing bubble and balances with the liquid inertia compressing the bubble, the bubble starts to bounce back. After subsequent collapses and rebounds, $R_{b}$ and $m_{b}$ finally become near zero, indicating an irreversible collapse of the bubble.

**Fig. 8:**
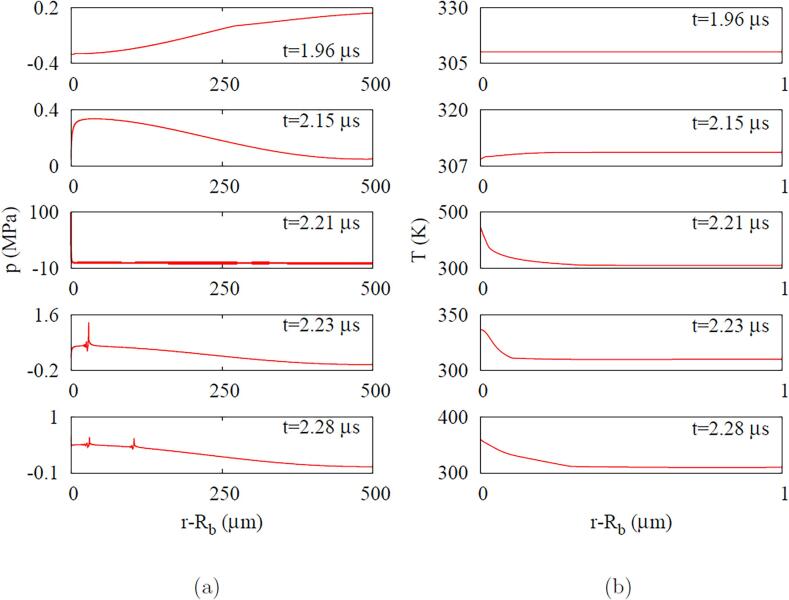
ADV at $f_{a}=2.25\;\text{MHz}$ and $P_{a,0}=0.50\;\text{MPa}$: (a) pressure and (b) temperature profiles near the bubble.

The liquid pressure and temperature profiles are plotted in Fig. 8. While the bubble compresses due to the positive pulsing, its pressure increases until the bubble bounces back at t=2.21μs (Fig. 8a). The bubble rebound generates a shock wave at t=2.23μs. The shock wave travels outward and attenuates to 37MPa,17MPa and 3.3MPa as it reaches r=1μm,r=2μm and r=10μm, respectively. This attenuation is nearly proportional to 1/r as described in Ref. [31]. Another shock wave is emitted at t=2.28μs after the second bubble rebounds. The liquid pressure changes in a large portion of the liquid region, but the liquid temperature variation is limited in a narrow region near the bubble, as depicted in Fig. 8b.

### 3.3 Effect of liquid compressibility on ADV

**Fig. 9:**
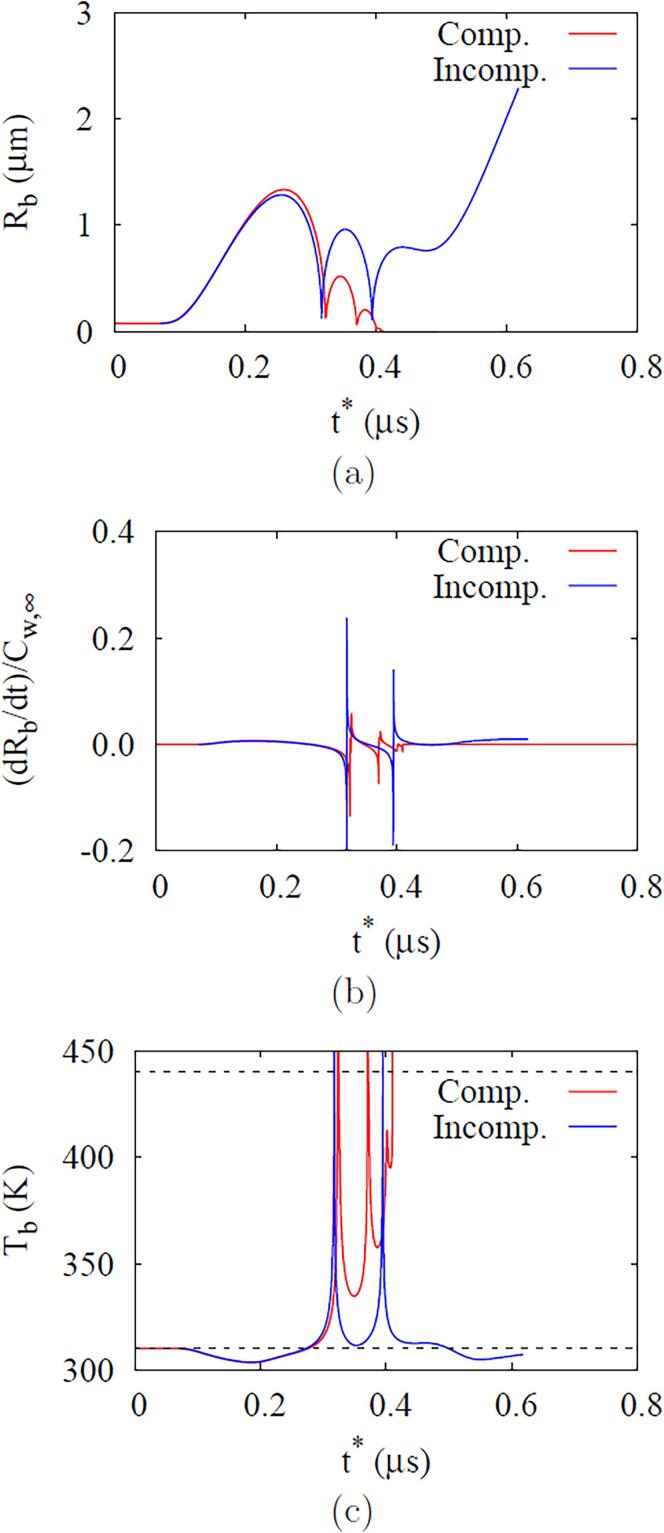
Effect of liquid compressibility on the ADV results at $f_{a}=2.25\;\text{MHz}$ and $P_{a,0}=0.50\;\text{MPa}$: (a) bubble radius, (b) surface velocity and (c) temperature. In the figure c, the upper and lower dashed lines indicate the critical temperature and ambient temperature, respectively.

In Fig. 9, we compare the ADV results for compressible and incompressible liquids. Considering that the pressure wave in the incompressible liquid case immediately reaches the droplet due to its infinite sound speed, and its magnitude is not amplified as $P_{a,0}=P_{a,L}$, the results are plotted against the relative time $t^{*}$ to have the same initial bubble growth pattern for the compressible and incompressible liquid cases. The discrepancy in both results is significant after the first bubble collapse and rebound. Compared to the results for the incompressible liquid case, the rebounding bubble has the first local maximum radius that is about 44.6% smaller for the compressible liquid case, the bubble surface speed becomes lower, and the time interval between two subsequent rebounds is reduced, which was also reported in Ref. [9]. Since the bubble radius is smaller during the collapse and rebound periods, $T_{b}$ remains higher in the compressible liquid case (Fig. 9c) and the bubble condenses and eventually disappears, whereas the bubble completely vaporizes at $t^{*}=0.68\;\mu \text{s}$ under the incompressible liquid condition.

**Fig. 10:**
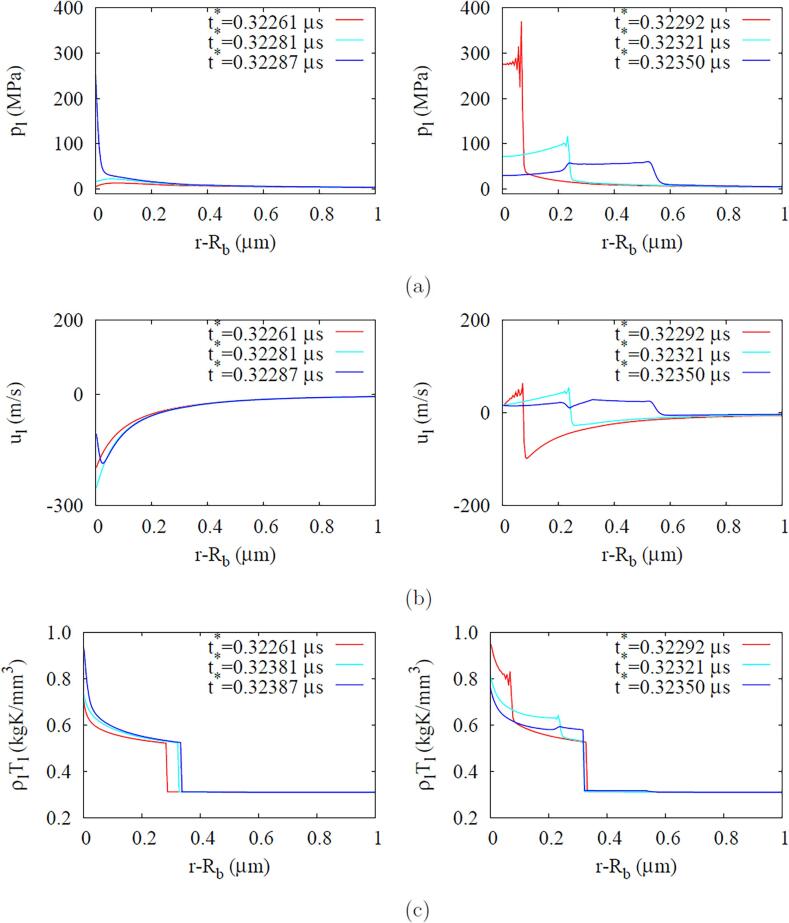
ADV in the compressible liquid case at $f_{a}=2.25\;\text{MHz}$ and $P_{a,0}=0.50\;\text{MPa}$: (a) liquid pressure, (b) velocity and (c) $\rho_{l}T_{l}$ profiles near the bubble during the first bubble collapse (left) and rebound (right) period.

**Fig. 11:**
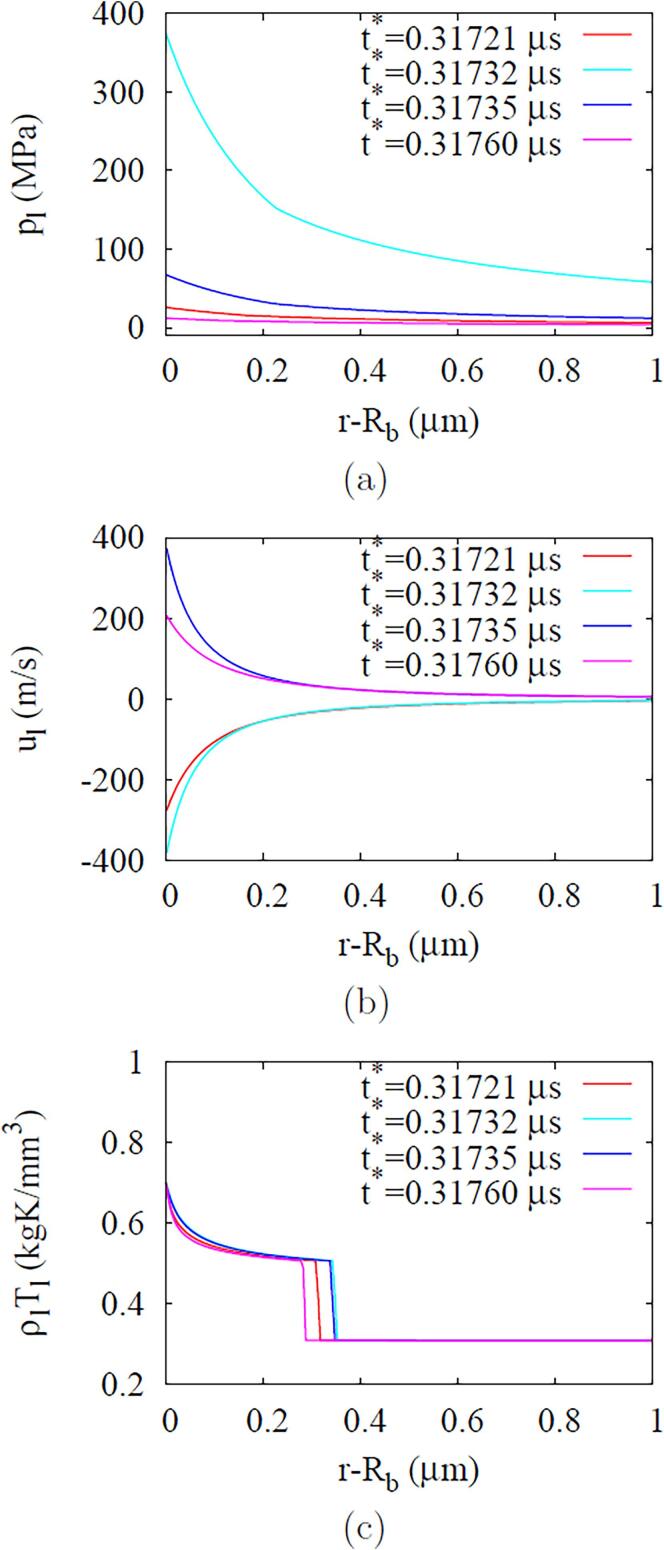
ADV in the incompressible liquid case at $f_{a}=2.25\;\text{MHz}$ and $P_{a,0}=0.50\;\text{MPa}$: (a) liquid pressure, (b) velocity and (c) $\rho_{l}T_{l}$ profiles near the bubble during the first bubble collapse and rebound period.

Fig. 10 shows the liquid pressure, velocity and $\rho_{l}T_{l}$ profiles near $r=R_{b}$ during the first bubble collapse and rebound period for the compressible liquid case. The profiles rapidly vary in a narrow liquid during the bubble collapse period, whereas their variations spread differently in a wider region with a shock wave traveling outward during the bubble rebound period. For the incompressible liquid case with no shock, however, the liquid pressure, velocity and $\rho_{l}T_{l}$ similarly change in reverse order during the bubble collapse and rebound periods, as plotted in Fig. 11.

To further clarify the effect of liquid compressibility on ADV, we analyze the temporal changes in the liquid energy while the bubble temperature $T_{b}$ is exceeds the critical temperature $T_{c}$ during the first collapse and rebound period of $t_{1}⩽t⩽t_{2}$, where the collapse time $t_{1}=0.3226\;\mu \text{s}$ and the rebound time $t_{2}=0.3249\;\mu \text{s}$ for the compressible liquid case, and $t_{1}=0.3172\;\mu \text{s}$ and $t_{2}=0.3175\;\mu \text{s}$ for the incompressible liquid case. Neglecting the contributions of heat transfer and viscous work to the liquid energy change during the collapse and rebound period at the supercritical state, the conservation equation of liquid energy can be written as

(31) $$\frac{\partial r^{2}\rho_{l}E_{l}}{\partial t}+\frac{\partial r^{2}u_{l}\rho_{l}E_{l}}{\partial r}=-\frac{\partial r^{2}p_{l}u_{l}}{\partial r}$$

where $E_{l}=c_{l}(T_{l}-T_{\infty })+u_{l}^{2}/2$. The integration of Eq. (31) from $r=R_{b}$ to $r=L^{\prime }$, which is chosen as $40R_{\mathrm{do}}$ or 20μm, yields

(32) $$\frac{d}{\mathrm{dt}}\int_{R_{b}}^{L^{\prime }}(r^{2}\rho_{l}E_{l})\mathrm{dr}=-{\left(r^{2}p_{l}u_{l}\right)}_{R_{b}}^{L^{\prime }}-r^{2}(u_{l}-\frac{{\mathrm{dR}}_{b}}{\mathrm{dt}}){\left(\rho_{l}E_{l}\right]}_{R_{b}}^{L^{\prime }}$$

Considering that the second term on the right hand side of the above equation is relatively negligible because $u_{l}={\mathrm{dR}}_{b}/\mathrm{dt}$ at $r=R_{b}$ and $E_{l}≃0$ at $r=L^{\prime }$, we have

(33) $${\mathrm{KE}}_{t}+\Delta \mathrm{IE}+\Delta \mathrm{FW}={\mathrm{KE}}_{t1}$$

where the liquid kinetic energy (*KE*), the internal energy (*IE*) and the flow work (*FW*) are defined as

(34) $$\mathrm{KE}=4\pi \int_{R_{b}}^{L^{\prime }}\frac{1}{2}\rho_{l}u_{l}^{2}r^{2}\mathrm{dr}$$

(35) $$\Delta \mathrm{IE}={\left(\mathrm{IE}\right)}_{t}-{\left(\mathrm{IE}\right)}_{t_{1}}=4\pi {\left[\int_{R_{b}}^{L^{\prime }}\rho_{l}c_{l}(T_{l}-T_{\infty })r^{2}\mathrm{dr}\right]}_{t_{1}}^{t}$$

(36) $$\Delta \mathrm{FW}=4\pi \int_{t_{1}}^{t}[{\left(r^{2}p_{l}u_{l}\right)}_{L^{\prime }}-{\left(r^{2}p_{l}u_{l}\right)}_{R_{b}}]\mathrm{dt}$$

**Fig. 12:**
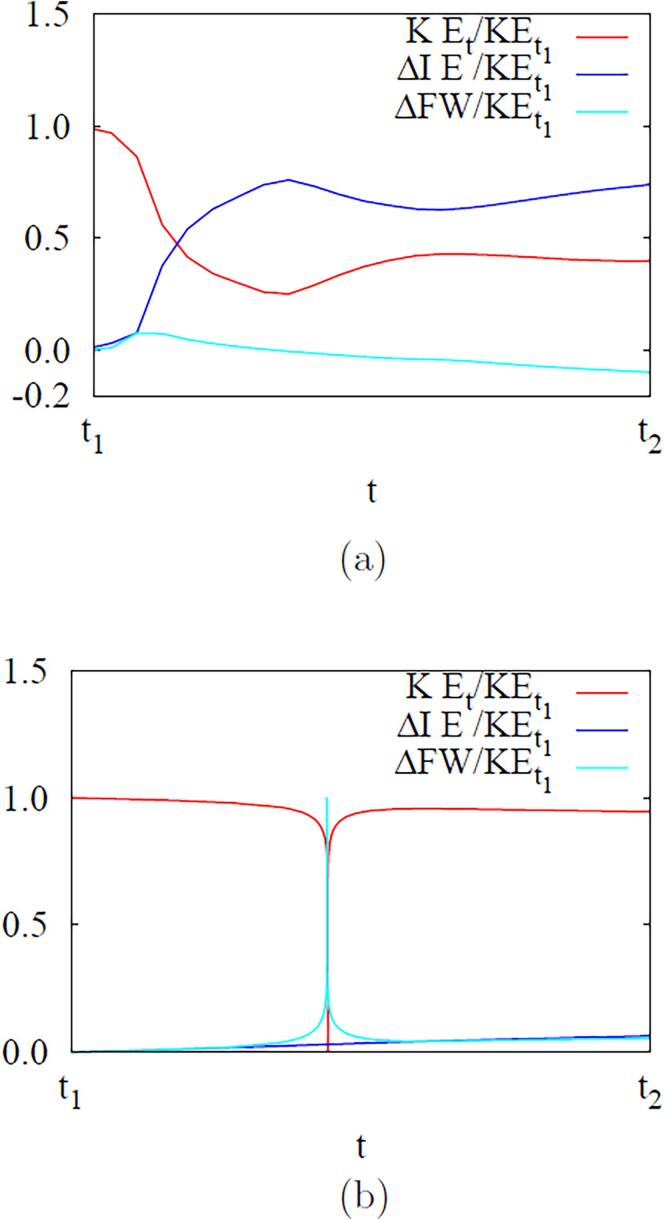
Effect of liquid compressibility on the energy variation of the surrounding liquid during the bubble collapse and rebound period (from $t_{1}$ to $t_{2}$) at $f_{a}=2.25\;\text{MHz}21$ and $P_{a,0}=0.50\;\text{MPa}$: (a) compressible and (b) incompressible liquid cases.

The results are plotted in Fig. 12. The loss of kinetic energy for the compressible liquid case during the bubble collapse and rebound is almost 60%, which is 6 times greater than for the incompressible liquid case. The loss of kinetic energy results in the amount of internal or thermal energy increase for both cases. For the compressible liquid case, the liquid internal energy is increases by 70% due to not only the bubble motion and shock wave but also the flow work of positive pressure pulsing during the bubble collapse and rebound period.

### 3.4 Effects of liquid compressibility and acoustic frequency

**Fig. 13:**
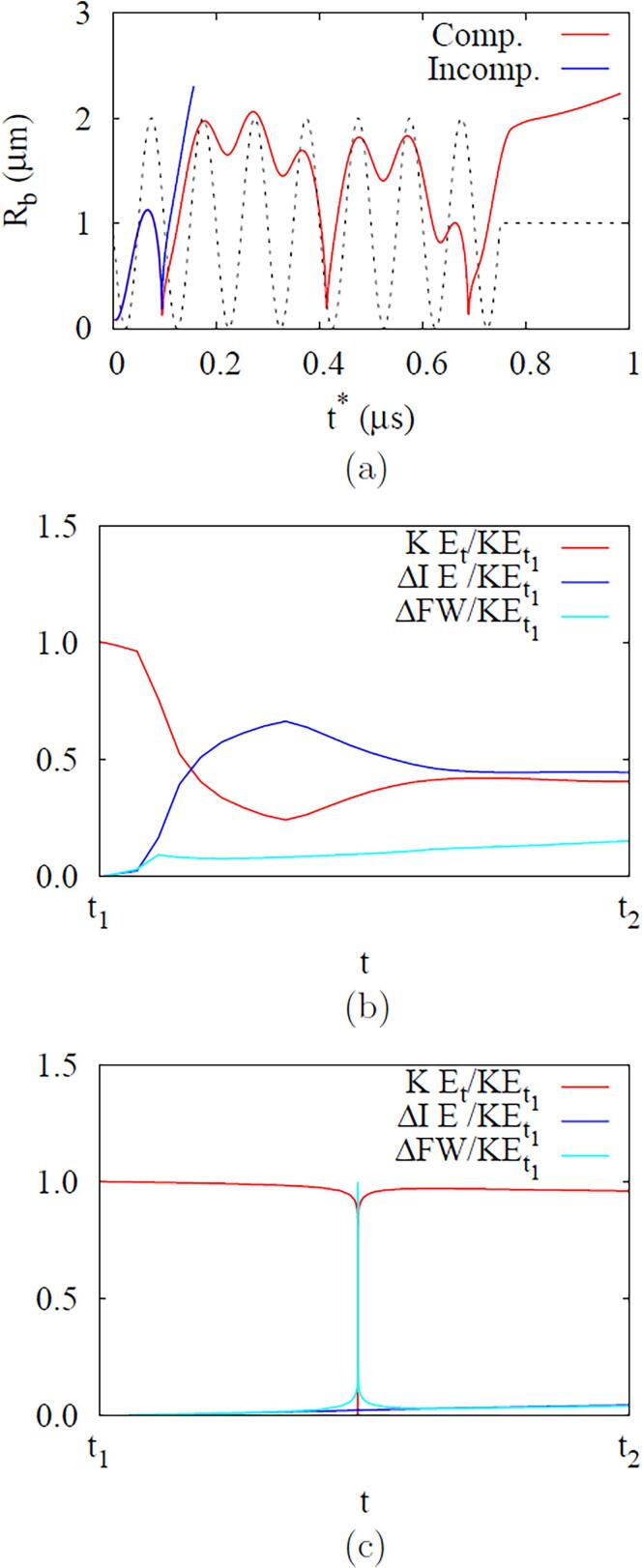
Effect of liquid compressibility on ADV at $f_{a}=10\;\text{MHz}$ and $P_{a,0}=1.57\;\text{MPa}$: (a) bubble radius variation and liquid energy variations during the bubble collapse and rebound period for (b) compressible and (c) incompressible liquid cases. In the figure a, the black dash line indicates acoustic pressure pulsing profile. In the figures b and c, $t_{2}-t_{1}$ are 2ns for the compressible liquid case and 0.263ns for the incompressible liquid case, respectively.

Fig. 13 shows the ADV results at a higher frequency of $f_{a}=10\;\text{MHz}$ and $P_{a,0}=1.57\;\text{MPa}$. Compared to the case of $f_{a}=2.25\;\text{MHz}$, the bubble radius varies more dynamically because the sign of pressure pulse changes rapidly with increasing $f_{a}$. When the loss of liquid kinetic energy during the first collapse and rebound period is evaluated as done in Section 3.3, it is 4.5% for the incompressible liquid case and increases to 60% for the compressible liquid case. The liquid internal energy for the compressible liquid case is increased by 43%, which is caused by the flow work of negative pressure pulsing in the bubble collapse and rebound period. The bubble vaporizes completely at $t^{*}=0.16\;\mu \text{s}$ for the incompressible liquid case, but the bubble under the compressible liquid condition undergoes multiple collapse and rebounds and then completely vaporizes 0.82μs later than in the impressible liquid case.

**Fig. 14:**
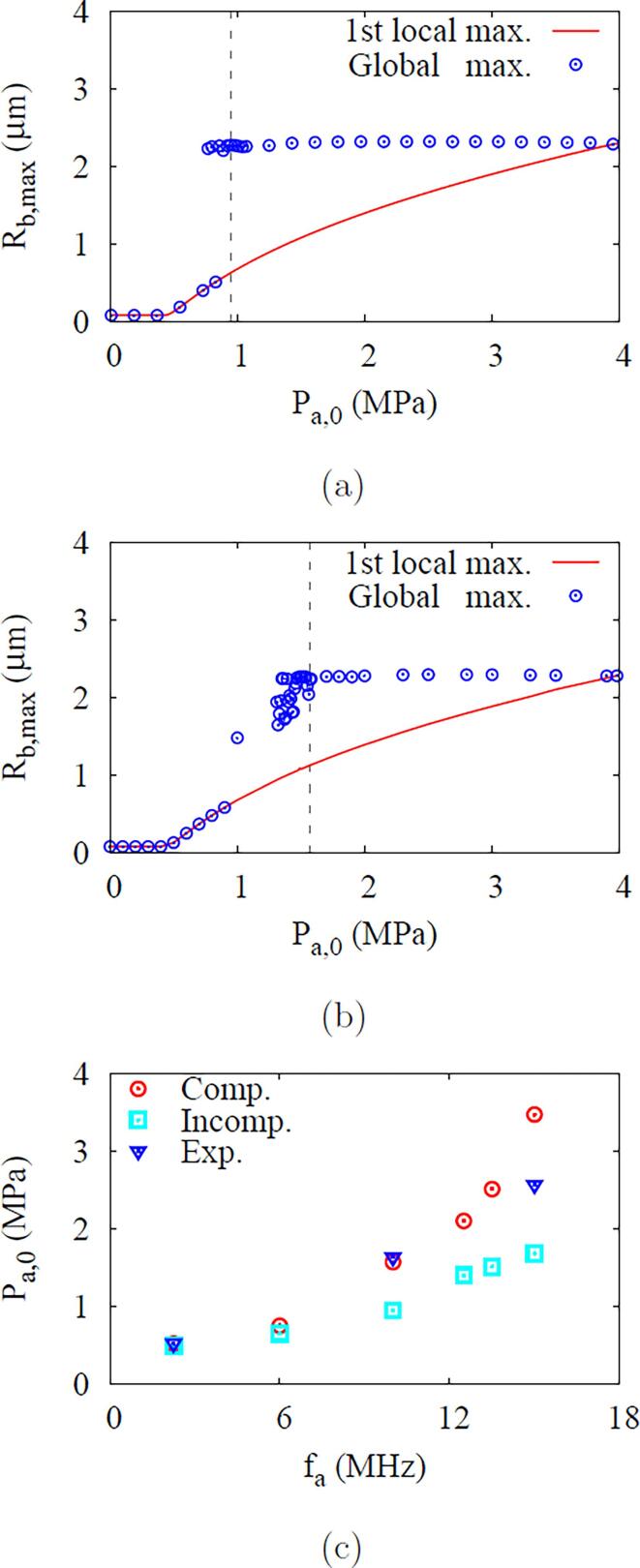
Effect of liquid compressibility on the ADV threshold: maximum bubble radi for the (a) incompressible and (b) compressible liquid cases at $f_{a}=10\;\text{MHz}$, and (c) ADV threshold depending on acoustic frequency. In the figures a and b, the vertical dashed lines indicate the ADV threshold.

Fig. 14a and b present the first local and global maximum bubble radii in ADV at $f_{a}=10\;\text{MHz}$. For the incompressible liquid case, the first local maximum bubble radius increases gradually with $P_{a,0}$, but the global maximum bubble radius increases abruptly near the ADV threshold of $P_{a,0}=0.95\;\text{MPa}$. The rapid bubble growth after collapse and rebound helps the bubble to completely vaporize, as described in Refs. [6], [7]. On the other hand, the global maximum bubble radius for the compressible liquid case gradually increases near the ADV threshold of $P_{a,0}=1.57\;\text{MPa}$, as seen in Fig. 14b, because the liquid compressibility inhibits the bubble growth during the bubble collapse and rebound, as depicted in Fig. 13.

The combined effects of liquid compressibility and $f_{a}$ on the ADV threshold are shown in Fig. 14c. The liquid compressibility is observed to increase the ADV threshold, especially as $f_{a}$ increases. The predicted ADV thresholds considering liquid compressibility are 0.52MPa for $f_{a}=2.25\;\text{MHz}$ and 1.57MPa for $f_{a}=10\;\text{MHz}$, which are 6% and 65% higher than for the incompressible liquid cases, respectively. The numerical predictions taking account liquid compressibility are more comparable to the experimental data except near $f_{a}=15\;\text{MHz}$. The experimental data at $f_{a}=15\;\text{MHz}$ is close to the computed ADV threshold at a slightly lower frequency of $f_{a}=13.5\;\text{MHz}$. This discrepancy may be due to the effect of phospholipid coating or encapsulation of the droplet, as investigated in the previous study [7], or the nonlinear effect of acoustic wave transmission occurring under high-frequency conditions, as described in Refs. [27], [32].

## 4 Conclusions

The effect of liquid compressibility on ADV of volatile microdroplets was numerically investigated by directly solving the conservation equations for the liquid droplet and surrounding water regions. The computations of acoustic pressure waves traveling in the compressible liquid regions showed that the magnitude of acoustic waves near the domain center is amplified in proportion to the acoustic frequency and inversely to the sound speed. The numerical results for ADV demonstrated that the bubble motion is significantly influenced by liquid compressibility during the bubble collapse and rebound periods. As the bubble compresses, the bubble pressure exceeds the critical pressure and rapidly rises to a very high pressure at the supercritical state without phase change. During the subsequent rebound period, the bubble generates a shock wave and the liquid region near the bubble loses about 60% of its kinetic energy under the compressible liquid condition, which is 6 times greater than under the incompressible liquid condition. This indicates that the liquid compressibility highly inhibits bubble growth during bubble collapse and rebound. As a result, the ADV threshold is higher for the compressible liquid case than for the incompressible liquid case, especially under high-frequency conditions. The numerical predictions of ADV thresholds are in better agreement with the experimental data, including the liquid compressibility effect.

## CRediT authorship contribution statement

**Sukwon Park:** Writing – original draft, Conceptualization, Software. **Gihun Son:** Writing – review & editing, Conceptualization, Methodology, Supervision.

## Declaration of Competing Interest

The authors declare that they have no known competing financial interests or personal relationships that could have appeared to influence the work reported in this paper.
